# Effect of Sr Doping on Structural and Transport Properties of Bi_2_Te_3_

**DOI:** 10.3390/ma14247528

**Published:** 2021-12-08

**Authors:** Yurii G. Selivanov, Victor P. Martovitskii, Mikhail I. Bannikov, Aleksandr Y. Kuntsevich

**Affiliations:** P.N. Lebedev Physical Institute of the RAS, 119991 Moscow, Russia; martovickijvp@lebedev.ru (V.P.M.); bannikov@lebedev.ru (M.I.B.)

**Keywords:** topological insulator, topological superconductivity, doping, thin films, molecular beam epitaxy, single crystals

## Abstract

Search for doped superconducting topological insulators is of prime importance for new quantum technologies. We report on fabrication of Sr-doped Bi${}_{2}$Te${}_{3}$ single crystals. We found that Bridgman grown samples have *p*-type conductivity in the low 10${}^{19}$ cm${}^{-3}$, high mobility of 4000 cm${}^{2}$V${}^{-1}$s${}^{-1}$, crystal structure independent on nominal dopant content, and no signs of superconductivity. We also studied molecular beam epitaxy grown Sr${}_{x}$Bi${}_{2-x}$Te${}_{3}$ films on lattice matched (1 1 1) BaF${}_{2}$ polar surface. Contrary to the bulk crystals thin films have *n*-type conductivity. Carrier concentration, mobility and *c*-lattice constant demonstrate pronounced dependence on Sr concentration *x*. Variation of the parameters did not lead to superconductivity. We revealed, that transport and structural parameters are governed by Sr dopants incorporation in randomly inserted Bi bilayers into the parent matrix. Thus, our data shed light on the structural position of dopant in Bi${}_{2}$Te${}_{3}$ and should be helpful for further design of topological insulator-based superconductors.

## 1. Introduction

Bismuth chalcogenides as topological insulators (TI) and narrow gap semiconductors represent a vast playground for novel physics and applications in the fields of thermoectricity [1], spintronics [2], unusual superconductivity [3], photodetectors [4], optical coatings [5] etc. Possible existence of Majorana fermions [6,7] and potential use in fault-tolerant quantum computing [8] draw a great interest in topological superconductors (TSC) [9]. Fundamental interest in search for materials that can display topological superconductivity (TS) is also supported by features such as zero bias conduction peak [10,11] and delocalized Andreev bound states [12].

Due to the strong spin–orbit coupling the superconductors derived directly from the topological insulators are promising candidates for TSCs. Binary bismuth-based TIs Bi${}_{2}$Se${}_{3}$ and Bi${}_{2}$Te${}_{3}$ both have tetradymite structure and become superconductors under high pressure [13,14] or due to the proximity effect [15] in close contact of TI with conventional superconductor. Still, doping is more straightforward and flexible approach to achieve superconductivity or to modify transport, optical and magnetic properties of parent TI.

A bulk topological superconductivity with critical temperature $T_{c}$ around 3 K was most intensively studied in the *n*-type doped 3D TIs of the bismuth selenide family M${}_{x}$Bi${}_{2}$Se${}_{3}$ (where M = Cu, Sr, and Nb) [3,10,11,16,17,18,19,20,21,22]. Only a few papers reported superconductivity derived from the p-type doped sister compound bismuth telluride A${}_{x}$Bi${}_{2}$Te${}_{3}$ (where A = Tl or Pd) [23,24,25,26,27]. More intriguingly, combination of two non superconductive materials (one of them bismuth telluride) demonstrates superconductivity in epitaxial Bi${}_{2}$Te${}_{3}$-FeTe heterostructures [28,29,30]. So, Bi${}_{2}$Te${}_{3}$ is an attractive platform for creating TSC. Indeed, one may expect to achieve superconductivity with novel appropriate dopant atoms. We could bargain for the hole conductivity by Fermi level tuning if Sr${}^{2+}$ would successfully act as an acceptor replacing Bi${}^{3+}$ in the crystalline matrix.

In our recent paper [31] we have grown Sr-doped Bi${}_{2}$Se${}_{3}$ thin films and bulk crystals with a wide range of Sr-content. The films differ from the bulk crystals in their structure, morphology and electrophysical properties. Replacempent of Se for Te may change the structure and properties of the films and bulk crystals in Te-based system. Novel results would also allow to shed light on the different behaviour of Sr dopant atoms in thin films and bulk crystals. Further thin-film technology optimization may open the door for manufacturing superconducting bismuth chalcogenides thin films, not yet achieved.

In this study, we report an attempt to achieve superconductivity in Sr-doped Bi${}_{2}$Te${}_{3}$ bulk single crystals and epitaxial thin films. Crystals were obtained by modified Bridgman method, and thin films were MBE deposited on (1 1 1) BaF${}_{2}$ substrates. We performed detailed structural and low temperature magnetotransport studies. While crystals have *p*-type conductivity, thin films demonstrate *n*-type transport. Structure of the films and the crystals differ drastically as well. In bulk crystals the correlation between Sr content and *c*-lattice parameter was not observed. In the epitaxial layers *c*-lattice parameter systematically grows with nominal Sr content, however this dependence is weaker than in Sr-doped Bi${}_{2}$Se${}_{3}$ films [31]. We employed grazing diffraction at the (1 0 1) reflection and revealed secondary phases containing Bi–Bi bilayers. Both in bulk crystals and thin films the superconductivity was not observed. Our comparative study reveals the different roles of Sr dopant in structure and electronic properties modification for Bi${}_{2}$Te${}_{3}$ crystals and films. We demonstrate, that transport and structural parameters are governed by Sr dopants incorporation into Bi bilayers between quintuple layers in the parent Bi${}_{2}$Te${}_{3}$ matrix.

## 2. Materials and Methods

### 2.1. Growth and Characterization Techniques

Single crystals with nominal compositions Bi${}_{2}$Te${}_{3}$, Sr${}_{x}$Bi${}_{2}$Te${}_{3}$, Sr${}_{x}$Bi${}_{2}$Te${}_{3+x}$ were grown using the modified Bridgman method similarly to Sr-doped Bi${}_{2}$Se${}_{3}$ [19]. The corresponding molar ratios of the elementary high-purity Sr (99,95%) Bi(99,999%) and Te(99,999%) were placed into quartz ampoule in the inert atmosphere of the glove box, evacuated and hermetically sealed. Material was synthesized by heating the tubes at 850–900 ${}^{\circ }$C for 24 h with periodic stirring. Single crystals were grown by a slow cooling of the melt from 750 down to 530 ${}^{\circ }$C at a rate of 2 ${}^{\circ }$C per hour. The samples were then annealed at 530 ${}^{\circ }$C for 24 h and quenched in cold water. An attempt to fix possible metastable structural phase (as was found for Cu-doped Bi${}_{2}$Se${}_{3}$[32]) revealed that samples quenched from slightly higher temperature (560 ${}^{\circ }$C) turned out to be polycrystalline ingots with the crystallites linear dimensions 0.3–1 mm.

Sr-doped Bi${}_{2}$Te${}_{3}$ films on (1 1 1) BaF${}_{2}$ substrates were fabricated in an MBE apparatus EP-1201, (EZAN, Chernogolovka, Russia) with a residual pressure $3\cdot 10^{-10}$ Torr in the growth chamber [31,33]. Atomic/molecular fluxes were generated from resistive effusion cells loaded with high purity elemental Te, Sr and binary Bi${}_{2}$Te${}_{3}$ compound, and provided growth rate of ∼0.3 nm/min. Fluxes were controlled by measuring beam equivalent pressure (BEP) from each cell. BEP flux ratio Te/Bi${}_{2}$Te${}_{3}$ of 3:1 was supported by maintaining stable cell temperatures of 250 and 470 ${}^{\circ }$C for Te and Bi${}_{2}$Te${}_{3}$, respectively. Unlike “ramp up” growth protocol used for Sr-doped Bi${}_{2}$Se${}_{3}$ films [31], here we followed a single step growth procedure [34] owing to the higher sticking coefficient of Te ad-atoms as compared to the Se flux. The substrate temperature in the present work was kept at 300 ${}^{\circ }$C. Initially, 2 nm thick Bi${}_{2}$Te${}_{3}$ buffer layer was deposited, followed by the growth of ternary Sr${}_{x}$Bi${}_{2-x}$Te${}_{3}$ film with thickness 26–35 nm. Molar composition *x* was regulated from 0.003 to 0.283 by increasing the Sr cell temperature from 266 to 375 ${}^{\circ }$C. Based on measured and approximated fluxes, generated by Sr and Bi${}_{2}$Te${}_{3}$ cells, we derived Sr concentration *x* in the layers as described in [31]. We used an additional cell with a BaF${}_{2}$ source for deposition of protective 40 nm thick BaF${}_{2}$ cap layer in the same growth chamber.

### 2.2. X-ray Diffraction

The X-ray diffraction(XRD) and X-ray reflection(XRR) measurements were carried out on Panalytical MRD Extended diffractometer with a hybrid monochromator (PANalytical, Almelo, The Netherlands), that is a combination of a parabolic mirror and a single crystal 2 × Ge(220) monochromator. We used triple crystal-analyzer 3 × Ge(220) to get high resolution (2θ/ω)-scanning curves for lattice parameter determinations. Thickness of the films was obtained from (006) Bragg peak diffraction fringes and/or from X-ray reflection (XRR) spectra.

For measurements of Sr concentration in single crystals EDX scans were performed using JSM-7001F microscope (JEOL Inc., Tokyo, Japan) with Oxford Instruments analyzer (Oxford Instruments NanoAnalysis & Asylum Research, High Wycombe, UK).

### 2.3. Transport Measurements

For transport measurements Hall-bar geometries were defined by scratching the films with needle, similarly to References [31,33]. The crystals were cut into rectangular shape as explained in Reference [35]. Samples were mounted on the holder and contact wires were attached with silver paint (contact resistance was typically below several hundreds Ohms for films and several tens Ohms for crystals). Low-temperature magnetotransport measurements were performed using a standard lock-in technique at frequencies 13–80 Hz and measurement current 0.5–1 μA (for films and 100 μA for crystals) to ensure the absence of overheating at the lowest temperatures. All measurements were performed in the temperature range 1.6 K–300 K using the Cryogenics dry CFMS-16 system (Cryogenic Ltd, London, UK). Perpendicular magnetic field was swept at constant temperatures (typically at 4.2, 77 and 300 K) from positive to negative value (typically 2 T). In order to compensate contact misalignment, the magnetoresistance (Hall resistance) data were symmetrized (antisymmetrized). Using the $\rho_{xx}\left(B\right)$ and $\rho_{xy}\left(B\right)$ dependencies we determined the carrier density and Hall mobility.

## 3. Results

### 3.1. Single Crystals

**Figure 1:**
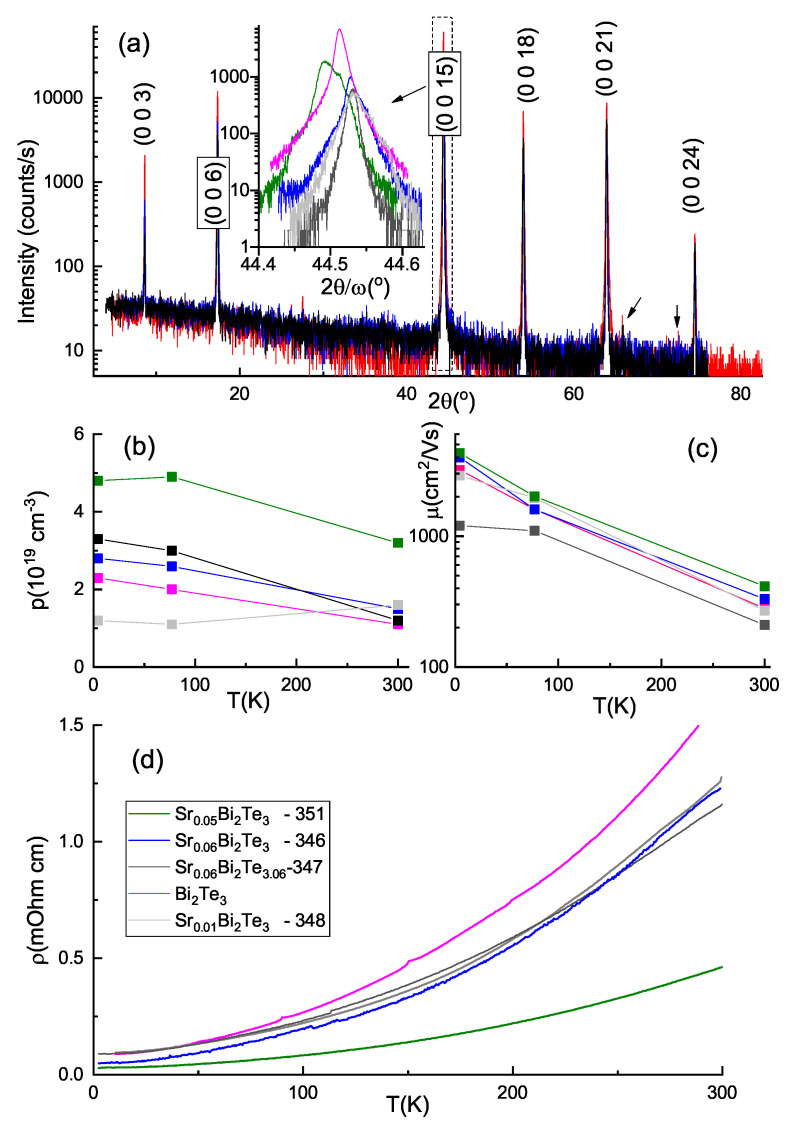
(**a**) 2θ XRD scans of the studied crystals. Insert of panel (**a**) shows 2θ/ω scans for precise determination of the *c*-lattice parameter; temperature dependencies of Hall-effect density (**b**), mobility (**c**) and resistivity (**d**).

The crystals cleaved easily along the basal plane and demonstrated mirror-like surfaces. Parameters of the studied samples are indicated in the insert of Figure 1d and marked by different colors. High-intensity XRD scans with a series of (003l) peaks (in hexagonal notations) are shown in Figure 1a. and display clear coincidence of the spectra for all the samples. If at low Sr content (sample 348) we note a complete spectra overlap with that of binary Bi${}_{2}$Te${}_{3}$, for higher nominal *x* composition (samples 346, 351) we register additional reflections shown by three arrows in Figure 1a. So, already at x=(0.05–0.06) the presence of the secondary phase admixture is reliably determined. We ascribe new peaks to formation of Bi${}_{1}$Te${}_{1}$ phase. An excess metallic Sr in the Bi–Te matrix could displace Bi atoms and provoke formation of additionally inserted Bi bilayers along with Bi${}_{\mathrm{Te}}$ antisite defects.

Interestingly, for sample 347 with the stoichiometry shifted towards Te excess (Te mole fraction of 3.06) the above three peaks become undetectable. Bearing in mind possible formation of cubic SrTe-related compound, we performed more detailed XRD study of the sample 347 (shown in Appendix A), and did observe another set of weak peaks, corresponding to the defective cubic phase with lattice constant 6.33 Å, virtually for Sr${}_{1-x}$Bi${}_{x}$Te compound. Despite the presence of the secondary phases, perfect crystallinity of the crystal body allowed a precise determination of the lattice parameters. Rather small deviations in the angular positions of the (0015) reflection (see insert Figure 1a) for different *x* values demonstrate that *c*-lattice parameter is not governed by the nominal Sr composition. We note, that prolonged annealing (72 instead of a regular 24 h) of the sample 351 resulted in broader (0 0 15) reflection (see insert Figure 1a) and the highest *p*-type carrier density (see Figure 1b) due to the increased concentration of the antisite defects. In-plane lattice constant was determined from asymmetric (205) reflections and demonstrated even more significant scatter in obtained values (see Appendix A). Fluctuations of the *c*- and *a*-lattice parameters could be explained by presence of a rather small dopant concentration in the matrix and nonuniform distribution of Sr atoms in the basal plane of the crystal. Moreover, we revealed a significant bending of the crystalline planes, implying that Sr atoms may be responsible for suppressing layered growth during crystallization, similar to vicinal growth observed for Sr-doped Bi${}_{2}$Se${}_{3}$ bulk crystals [36]. Contrary to the Sr${}_{x}$Bi${}_{2}$Se${}_{3}$ [17,31], no correlation between Sr content and lattice parameters was revealed. Thus, Sr atoms in the Bi–Te system do not enter crystalline structure above some limited *x* (well below 0.01) value during the melt growth and promote admixture of the secondary phases.

Transport parameters do not show correlation with *x* as well. So, despite obtaining in telluride system tempting p-type conductivity, the superconductivity was not observed in bulk Sr${}_{x}$Bi${}_{2}$Te${}_{3}$ crystals.

### 3.2. Structural Results on Thin Films

**Figure 2:**
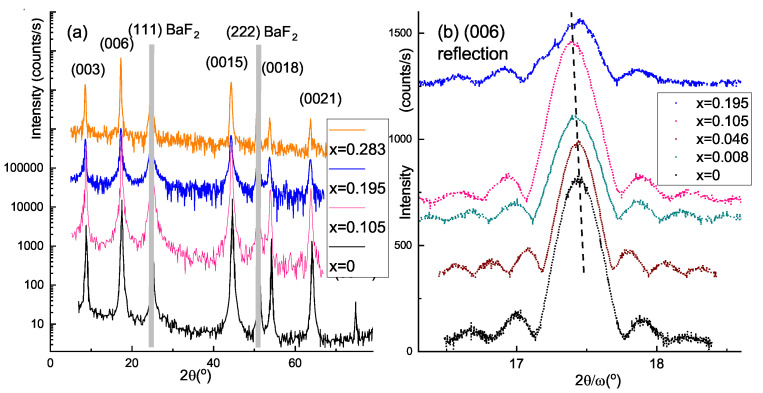
XRD scans for the epitaxial films with different Sr content *x*, indicated in the panel. (**a**) 2θ scans. (**b**) 2θ/ω XRD scans on (006) reflection for films with different *x* content increasing from bottom to top, indicated in the panel. The scan curves are shifted vertically for clarity.

Representative set of the 2θ-scans for thin films with different Sr content ($x_{\mathrm{Sr}}$ is listed in Table 1) is shown in Figure 2a. Intense (111) and (222) reflections from the BaF${}_{2}$ substrate are marked by gray vertical bars. Regular (003l) reflections from the films give evidence for the growth of crystallographically ordered and highly oriented layers with the (001) plane parallel to the (111) cleavage plane of the BaF${}_{2}$ substrate. With the increase of Sr concentration *x* from 0 to ∼0.2 the (003l) peak positions shifted to the lower values (with relative magnitude increase less than 0.3%), and the peaks get wider less than a factor of 3. Observed modest spectra modifications reflect survival of high crystallinity along with insignificant structural disorder (see variations in $c_{\left(0\;0\;15\right)}$ and $\Delta \omega_{\left(0\;0\;15\right)}$ in Table 1). While for sample with the highest Sr content (x=0.283) changes are more pronounced, still no additional peaks and no indication of the secondary phases were detected.

**Figure 3:**
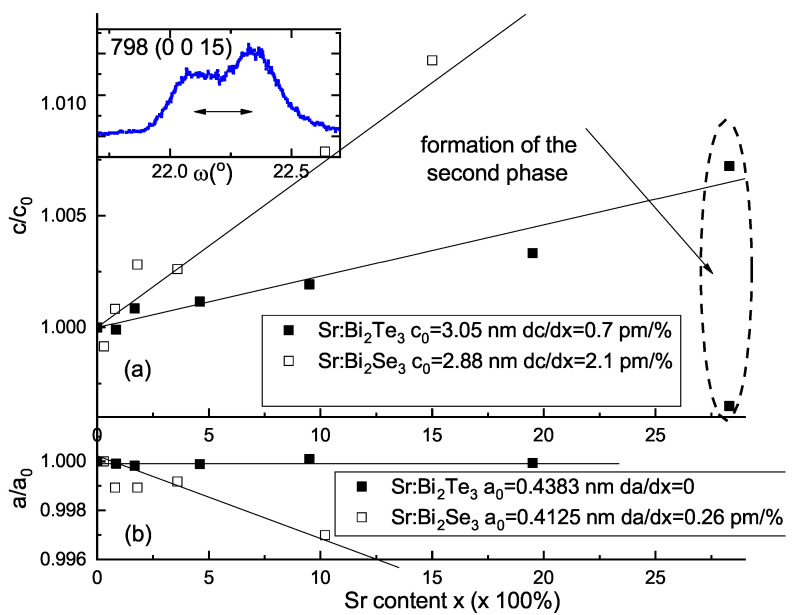
(**a**) Summary of the *c*-axis lattice parameter value measured on (0015) XRD reflection as a function of *x* for ∼25 nm thick Sr-doped Bi${}_{2}$Te${}_{3}$ films (black filled symbols). For comparison we show the data for Sr-doped Bi${}_{2}$Se${}_{3}$ by empty symbols [31]. Straight lines show approximations of c(x) dependencies for telluride and selenide-based thin films with the slopes ≈0.8 pm/% and ≈2.05 pm/%, respectively, (points, corresponding to second phase formation are not used for approximation). The insert shows XRD indication of nonuniformity (rocking curve at (0015)-reflection) of the film with the largest *x*-value. (**b**) Summary of the *a*-axis lattice parameter value for the same samples with the similar linear fits.

Interestingly, for all Sr doping levels we observe intensity fringes near the central (006) peak (see Figure 2b). Even two split peaks shown in the insert of Figure 3a, both produce well defined Bragg satellite resonances (not shown here) and each correspond nearly to the same film thickness. This observation also indicates that morphology and crystallinity of the films do not degrade significantly with *x*, contrary to Sr-doped Bi${}_{2}$Se${}_{3}$-films [31]. The period of the fringes might be used for measuring the film thickness [37], $L=\frac{\lambda }{2\times (\sin\omega_{2}-\sin\omega_{1})}$, where $\omega_{1}$ and $\omega_{2}$ are the positions of the satellite maxima, and λ is the X-ray wavelength (in our case 1.5406 Å). These values for the studied films are listed in the “d” column of Table 1, and are in good agreement with the thickness obtained with XRR data.

Figure 3 compares structural data for Sr-doped Bi${}_{2}$Te${}_{3}$ and Bi${}_{2}$Se${}_{3}$ thin films. Figure 3a shows the doping level dependencies of the normalized $c/c_{0}$- and $a/a_{0}$-lattice parameters, where ($c_{0}$, $a_{0}$) are binary Bi${}_{2}$Ch${}_{3}$ lattice constants, determined from (0015) and (205) reflections, respectively. Data for selenide system are taken from Reference [31]. In the composition range between x=0 and ∼0.2 for Sr-doped Bi${}_{2}$Te${}_{3}$ *c*-lattice parameter is linear and weakly dependent on *x*. The rate of *c*-lattice constant as a function of *x* in selenides is nearly a factor of 4 faster than in telluride system, pointing that Sr location sites in two sister compounds are rather different. Anion radius of Te${}^{2-}$ in octahedral surrounding (2.21 Å) is larger than that for Se${}^{2-}$ (1.98 Å), and observed slope for tellurides may reflect two scenarios: larger Sr${}^{2+}$ cation (1.18 Å) replaces Bi${}^{3+}$ cation (1.03 Å) in the Bi${}_{2}$Te${}_{3}$ structure and arranges ordered intercalated atoms in the van der Waals gap. With higher Sr content, for film 798, the rocking curve recorded at (0015) reflection turned out to be split (see insert of Figure 3a), revealing the admixture of the second phase. We conclude, that observed phase separation at x∼0.28 corresponds to the solubility limit of the Sr dopant in Bi${}_{2}$Te${}_{3}$ matrix under described MBE growth conditions. In plane *a* lattice constant for Sr-doped Bi${}_{2}$Te${}_{3}$ films (Figure 3b) is insensetive to the composition variation in contrast to the selenide system. These observations imply that in telluride matrix incoporation of Sr dopant atoms facilitate formation of specific atomic arrangement in the lattice (see Discussion section) in addition to producing substitutional point defects.

**Figure 4:**
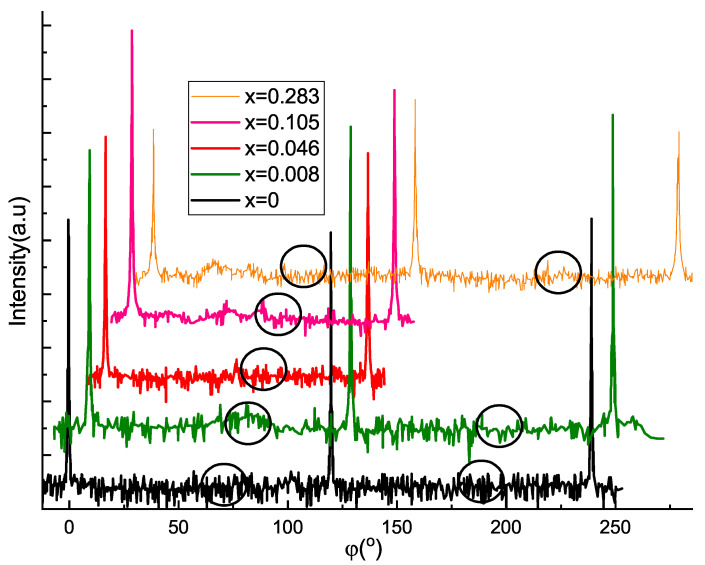
ϕ Scan of the [001] axis on (1010) reflection for films with different *x*. Circles denote angular positions of possible (not detected) twin domains. The scans are shifted along *x* and *y* axes for clarity.

Another notable distinction between Sr-doped Bi${}_{2}$Te${}_{3}$ and Bi${}_{2}$Se${}_{3}$ thin films on (111) BaF${}_{2}$ substrate is significant difference in the domain structure. Indeed, telluride ϕ-scans, shown in Figure 4, display purely single domain film structure (periodic 60${}^{\circ }$ peaks are not observed) in the whole range of the studied Sr concentrations up to *x*∼0.28. While phase separation takes place at comparable doping levels in telluride (*x*∼0.28) and selenide (*x*∼0.26) systems, Sr${}_{x}$Bi${}_{2-x}$Se${}_{3}$ films get completely twinned (see. Figure 6 of Reference [31]) already at x=0.018. Moreover, in selenide sample with maximal Sr content $60^{\circ }$ twins are accompanied with $30^{\circ }$ rotational domains. Twin domains strongly affect properties of the Bi${}_{2}$Ch${}_{3}$ family. Recently we demonstrated twin-free MBE growth for binary Bi${}_{2}$Te${}_{3}$ films on perfectly lattice matched (111) BaF${}_{2}$ substrates [38]. It turned out now that suggested approach preserves non twinned structure even for heavily Sr-doped crystals. We believe, that it is stability of in-plane lattice constant for the whole range of Sr concentration (Figure 3b), that allows to preserve single domain growth.

### 3.3. Transport Measurements on Thin Films

**Figure 5:**
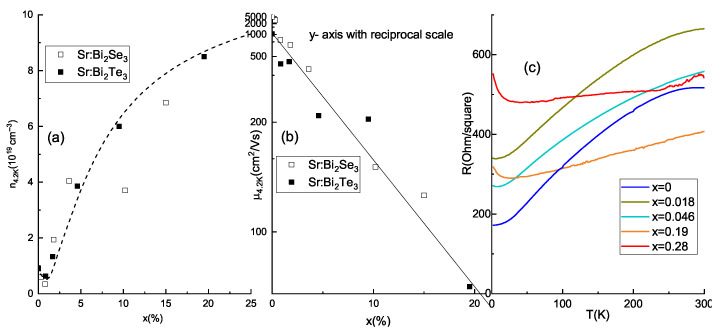
Transport data summary. Hall carrier density (panel (**a**)) and mobility (panel (**b**)) measured at T=2 K as a function of doping level for ∼30 nm thick films Bi${}_{2}$Te${}_{3}$-based films (black squares). Empty squares correspond to Bi${}_{2}$Se${}_{3}$-based films from Reference [31] The line in panel (**a**) is a guide for the eye, the dashed lines in panel (**b**) shows approximately linear *x*-dependence interpolation of the inverse mobility. (**c**) Temperature dependencies of the resistivity for the representative thin films.

Transport properties also show up systematic dependencies on *x*. All films have metallic type resistivity ($\frac{d\rho }{dT}>0$) in wide range of temperatures. The value of the resistivity per square tends to enhance progressively with Sr doping level for all temperatures. The residual-resistance ratios (RRR), defined as ratios between the resistance at 300 K and minimal resistance at low *T*, are summarized in Table 1. RRR ranges from 1.4 to 2.2 for most of the ∼25 nm thick Sr-doped films. This fact indicates the similar scattering mechanisms in all samples. In some films we observe the minimum of ρ and its low-*T* upturn (see Figure 5c). This minimum is believed to be caused by e-e interaction, similarly to numerous observations on undoped Bi${}_{2}$Se${}_{3}$ films [33].

Figure 5 collates free carrier density and mobility in the Sr-doped Bi${}_{2}$Te${}_{3}$ (filled boxes) and Bi${}_{2}$Se${}_{3}$ films (empty boxes), respectively. As obtained from the sign of the Hall effect, all films are *n*-type. Importantly, after some drop around *x*∼0.01, carrier density increases with *x* for both compounds, with more smooth behavior in telluride system, reflecting superior structural quality along with more uniform and reproducible doping process in Sr-doped Bi${}_{2}$Te${}_{3}$. Low-*x* drop indicates that for *x* below ∼0.01 Sr${}^{2+}$ substitution for the Bi${}^{3+}$ works as a dominant doping mechanism, as discussed in detail in [31]. Sr atoms mostly act as scattering centers and mobility is inversely proportional to *x*.

For x>0.02 the doping efficiency of Sr in both materials is very small (compare n(x) and dotted straight lines in Figure 8 of Reference [31]). It proves that (i) not each Sr atom act as a donor (moreover, the larger the value of *x* the weaker the doping effect, and carrier density saturates); (ii) At the same time the number of scattering centers (inverse mobility, see Figure 5b) grows roughly linearly with *x*. We believe, that Sr atoms in the telluride lattice act at least in two ways simultaneously: in one position (substitution of Bi) they act as acceptors, whereas in the other point defect positions they may act as donors. It is also probable (see Discussion section) that Sr promotes formation of Bi–Bi bilayers, also acting as donors. Anyway the doping efficiency of Sr is very small. Mobility (Figure 5b) decreases with *x* for both selenide- and telluride-based compounds.

## 4. Discussion

In our bulk crystals promising *p*-type conductivity was obtained. However, contrary to Tl- and Pd -doped Bi${}_{2}$Te${}_{3}$[23,24,25,26,27], we detect no signs of superconductivity. Our melt-grown Sr-doped Bi${}_{2}$Te${}_{3}$ bulk single crystals contained two different minority phases: layered (trigonal or rhombohedral) Bi${}_{x}$Te${}_{y}$ and cubic Sr${}_{1-x}$Bi${}_{x}$Te. So further increase of the Sr content is limited. Therefore we can not move the Fermi level deeply in the valence band thus increasing the density of states. We expect that variation of growth strategy and the use of different starting precursors (e.g., binary compounds instead of elements) may help to overcome this issue.

Our thin films have *n*-type of conductivity. Contrary to bulk crystals, they do not suffer from the admixture of the secondary cubic phase. In addition, Sr-doped Bi${}_{2}$Te${}_{3}$ films preserve high structural perfection at least to *x*∼0.2, when the carrier density reaches about 10${}^{20}$ cm${}^{-3}$, i.e., density typical for superconducting doped *n*-type Bi${}_{2}$Se${}_{3}$ materials. However this film are not superconductive. A precise knowledge of the Sr atoms location would be helpful to understand the key structural factor responsible for superconductivity in doped TI.

An ordered stacking of Te1–Bi–Te2–Bi–Te1 quintuple layers (QLs) and Bi–Bi bilayers (Bi BLs) building blocks may form bulk crystals of the (Bi${}_{2}{)}_{m}($Bi${}_{2}$Te${}_{3}{)}_{n}$ adaptive series [39]. In binary Bi${}_{2}$Te${}_{3}$ layers grown by MBE, excess BLs randomly inserted between the QLs were also observed [34,40,41]. Depending on the Te deficient flux and other growth conditions, and despite a statistical distribution of the additional Bi BLs, various phases such as Bi${}_{4}$Te${}_{5}$[40], Bi${}_{4}$Te${}_{3}$[41] and Bi${}_{1}$Te${}_{1}$[34] were identified in thin epitaxial films. More interestingly, MBE grown Mn-doped bismuth telluride thin films reveal the crystalline phase in which several Bi${}_{2}$Te${}_{3}$ quintuple layers were separated by Bi BLs [42].

Under adopted growth conditions in this paper, stoichiometric Bi${}_{2}$Te${}_{3}$ epi-layers are obtained with no signs of Bi–BLs, as evidenced by angular position of narrow diffraction peaks and supported by a rather high carrier mobility for sample with x=0. As compared with Eu-doped Bi${}_{2}$Te${}_{3}$[43] or Sr-doped Bi${}_{2}$Se${}_{3}$ films [31], our Sr${}_{x}$Bi${}_{2}$Te${}_{3}$ layers do not suffer from progressing structural disorder with doping level. On the contrary, as evidenced from (0 0 6) reflection thickness fringes (Figure 2b), moderate broadening of diffraction peaks (see Figure 2a along with column $\Delta \omega_{\left(0015\right)}$ of Table 1) and free carrier mobility (Figure 5b) in Sr${}_{x}$Bi${}_{2-x}$Te${}_{3}$ layers high crystallinity is preserved for *x* up to 0.2. These facts along with composition insensitive in-plane lattice parameter *a* allow us to assume that Sr incorporation in Bi${}_{2}$Te${}_{3}$ matrix favours Bi BLs to form between two consecutive Bi${}_{2}$Te${}_{3}$ QLs inside the van der Waals gap. Moreover, Sr dopant atoms are predominantly incorporated in this distributed Bi–Bi network. Sr as a one of the most active chemical element in the periodic Table may easily bind Te atoms and provoke Te-deficient growth conditions. Even as small deviation from stoihiometry as −0.02 (Bi${}_{2}$Te${}_{2.98}$) may produce 10% of Bi BLs in the film [44]. When increasing Sr content, the structure should change from pure Bi${}_{2}$Te${}_{3}$ phase through intermediate Bi${}_{x}$Te${}_{y}$ towards the Bi${}_{1}$Te${}_{1}$ phase, in which Bi–Bi BLs occurs periodically after each pair of QLs Te–Bi–Te–Bi–Te.

As it was previously demonstrated, XRD analysis is capable of detecting Bi interlayers in bismuth telluride matrix [45,46]. However, symmetrical (003l) XRD reflections can not reveal single layer of the phases BiTe or Bi${}_{3}$Te${}_{4}$ with the period 24 Å and 41.5 Å, respectively, due to broadened signal. It is necessary therefore to use a grazing diffraction in order to maximally elongate the path of the beam through the thin film. (101) reflection of Bi${}_{2}$Te${}_{3}$ is the most siutable for this purpose, because the sample plane has an angle $\psi =82.9^{\circ }$ with respect to the normal orientation, that corresponds to ∼8 times path elongation. Reflections (101) for the BiTe and Bi${}_{3}$Te${}_{4}$ phases will be located near the Bi${}_{2}$Te${}_{3}$ one. These reflections have maximal intensity for slightly different values of ψ (see Table 2), that allows systematic search of these phases.

**Figure 6:**
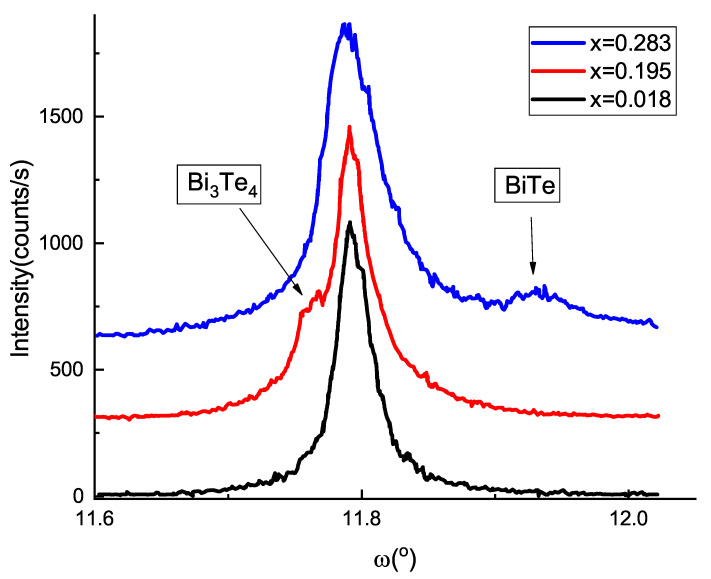
Rocking curves at (101) reflections for three different compositions demonstrating formation of Bi bilayers in the structure of the films.

Rocking curves recorded at the (101) peak at $\psi =82.9^{\circ }$ for three samples with increasing Sr content are shown in Figure 6. Vertical arrows indicate peaks corresponding to secondary phases. Sample with x=0.018 demonstrates a pure Bi${}_{2}$Te${}_{3}$ phase yet. When Sr composition is increased to x=0.195 the structure transforms into (Bi${}_{2}$Te${}_{3}$ + Bi${}_{3}$Te${}_{4}$) phase admixture. Further, with x=0.293 Bi${}_{2}$Te${}_{3}$ matrix is converted to Bi${}_{2}$Te${}_{3}$ + BiTe. So, we clearly demonstrated the presence and increase of Bi BL content with Sr composition in our thin films. The positions of the peaks for both BiTe and Bi${}_{3}$Te${}_{4}$ are shifted with respect to the estimated values. This is due to higher thermal expansion coefficients BaF${}_{2}$ substrate with respect to a film. After the growth therefore the film is contracted in the basal plane when cooled down to room temperature. This effect was not taken into account in Table 2, where room temperature lattice parameters are given for single-crystalline phases.

It was demonstrated previously [45] that Bi BLs in Bi${}_{2}$Te${}_{3}$ films act as electron donors, producing electron concentration well above 10${}^{20}$ cm${}^{-3}$. At the same time mobility was found to drop 50 times (down to 30 cm${}^{2}$V${}^{-1}$s${}^{-1}$) due to the asynchronous incorporation of the Bi doublets across the surface during growth. So, detected Bi BLs (Figure 6) could explain transport and structural features observed in our films.

## 5. Conclusions

In our paper, we report an effort to get superconducting Sr-doped Bi${}_{2}$Te${}_{3}$ bulk crystals and epitaxial thin films. While Bridgman grown Sr${}_{x}$Bi${}_{2}$Te${}_{3}$ single crystals have *p*-type conductivity, the parent compound films demonstrate *n*-type transport. None of the studied samples demonstrated superconductivity down to 1.6 K. In thin films Sr atoms induce formation of Bi BLs and predominantly are located within arranged Bi–Bi network. We believe, that information on exact location of Sr dopants is the key factor to resolve the problem and achieve superconducting phase. At present time even in superconducting selenides M${}_{x}$Bi${}_{2}$Se${}_{3}$ (where M = Sr, Cu, Nb) matrix crystallographic dopant positions are not reliably established [47]. Our results should be helpful for the control of Bi${}_{2}$Te${}_{3}$ properties by doping and indicate that further studies of doped TI are needed to create novel topological superconductors. 

## Figures and Tables

**Table 1:** Summary of sample parameters. The amount of Sr in Bi${}_{2}$Te${}_{3}$ films x${}_{Sr}$ was calculated from the flux ratios. Thickness *d* and parameters a,c were determined from XRD. Carrier density(*n*) and mobility (μ) were obtained from low-field and lowest temperature Hall measurements. Residual-resistivity ratio (RRR) was calculated as room temperature resistivity ($R_{T=300K}$) ratio to the low temperature resistivity minimum.

Sample	*x*	d (nm)	$c_{\left(0\;0\;6\right)}$(Å)	$c_{\left(0\;0\;15\right)}$(Å)	*a*(Å)	$△\omega_{\left(0\;0\;15\right)}$	RRR	n (cm${}^{-3}$)	μ (cm${}^{2}$/Vs)
773A	0	27.8	30.4839	30.5053	4.3835	0.088	3	$1.3\times 10^{19}$	1020
774	0.0084	34.7	30.4890	30.5020	4.3831	-	2.14	$6.3\times 10^{18}$	428
799	0.0168	31	-	30.5313	4.3827	0.189	1.97	$1.33\times 10^{19}$	447
786A	0.046	28	30.5270	30.5404	4.3830	0.180	2.2	$3.86\times 10^{19}$	213
787A	0.105	26	30.5534	30.5640	4.3839	0.209	1.828	$6\times 10^{19}$	206
794A	0.195	27	-	30.6065	4.3832	0.253	1.4	$8.9\times 10^{19}$	80
798A	0.283	29	-	30.592	4.3866	2 peaks	1.1	$3\times 10^{20}$	13

**Table 2:** Lattice constants and parameters of the relevant closely-placed asymmetrical reflection used to detect admixture of Bi BL-mediated phases. ψ is indicated with respect to (001)—direction.

Composition	*c* (Å)	*a* (Å)	θ(${}^{\circ }$)	ψ (${}^{\circ }$)
Bi${}_{2}$Te${}_{3}$	30.6065	4.3835	11.799	82.93
BiTe	24.002	4.402	11.806	80.98
Bi${}_{3}$Te${}_{4}$	41.50	4.416	11.670	84.73

**Figure A1:**
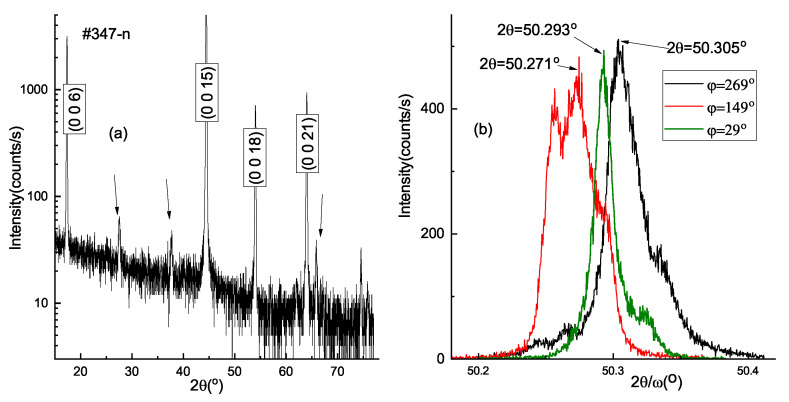
High resolution XRD data for sample 347. (**a**) 2θ scan of the selected piece of the crystal. The arrows indicate the peaks demonstrating the presence of the cubic phase. (**b**) 2θ/ω scans at the asymmetric (205) reflection collected for three polar angles (indicated in the panel).

## Data Availability

The data presented in this study are available in the article. On request the data in raw format could be requested from the corresponding authors.

## Abbreviations

The following abbreviations are used in this manuscript:

XRD	X-ray diffraction
XRR	X-ray reflection
MBE	Molecular beam epitaxy
TSC	Topological superconductivity
3D	Three-dimensional
TI	Topological insulator
BEP	beam equivalent pressure
CFMS	Cryomagnetic measurement system
BL	bilayer
QL	quintuple layer

## Appendix A. XRD Determination of Admixture Phases and Lattice Parameters in Single Crystals

We selected a piece of 347 sample and performed 2θ scan (see Figure A1a). There are three additional peaks (marked by the arrows). Two of them at $2\theta =37.69^{\circ }$ and $2\theta =65.95^{\circ }$ could be interpreted either as BiTe (0010) and (0017) reflections or as (220) and (420) of the cubic phase SrTe (a=6.47 Å) with sligtly reduced value of the lattice parameter (6.30–6.33 Å). At the same time the peak at $2\theta =28.03^{\circ }$ corresponds to the strongest (200) reflection of SrTe with lattice parameter a=6.36 Å. This peak is absent in BiTe and Bi${}_{3}$Te${}_{4}$ phases. Our data are thus indicative for the inclusions of the second phase Sr${}_{1-x}$Bi${}_{x}$Te with small value of *x* that modifies the lattice constant.

*c*-lattice parameter in tetradymite structure materials can be straightforwardly determined from the 2θ/ω scans at intensive symmetrical reflections (either (006) or (0015), see examples in Figure 1 and Figure 2 and Table 1).

For these precise measurements a triple Ge(220)×3 crystal-monochromator was used that provided 12” angular resolution. Prior to measurements zero position of the diffractometer was set using (004) reflection of the ultra pure silicon crystal with the well known lattice parameter $a_{Si}=5.43105$ Å. In order to determine the in-plane lattice parameter *a* a grazing diffraction geometry was used at (205) reflections with inclination angle $\psi =72.5^{\circ }$. These reflections are three-fold repeated and should be reproduced as the sample is rotated by Δϕ=120${}^{\circ }$ about the axis, perpendicular to basal plane.

Angular position of these reflections in 2θ/ω scans together with previously measured *c*-lattice parameter allows to determine *a*-parameter. Figure A1b shows 2θ/ω-scans collected for the same sample (347) in three different directions. The values of *a*-parameter calculated with known c=30.493 Å range from 4.3835 Å to 4.3865 Å. In other words this crystal has in-plane structural distortions.
